# Does endometrial compaction before embryo transfer affect pregnancy outcomes? a systematic review and meta-analysis

**DOI:** 10.3389/fendo.2023.1264608

**Published:** 2023-11-14

**Authors:** Xiao-Tong Chen, Zhen-Gao Sun, Jing-Yan Song

**Affiliations:** ^1^ The First Clinical College, Shandong University of Traditional Chinese Medicine, Jinan, China; ^2^ Reproductive and Genetic Center, The Affiliated Hospital of Shandong University of Traditional Chinese Medicine, Jinan, China

**Keywords:** endometrial compaction, transvaginal ultrasound, *in vitro* fertilization, frozen-thawed embryo transfer, endometrial thickness

## Abstract

**Objective:**

There is no clear evidence of clinical significance of endometrial compaction, which can be measured by a reduction in endometrial thickness (EMT) during the follicular-luteal transition before the day of embryo transfer. In this study, we aim to determine whether endometrial compaction has an effect on *in vitro* fertilization (IVF) success.

**Method(s):**

We searched PubMed, Cochrane, Embase, and Web of Science electronic databases for studies published in English up to March 2023. Heterogeneity between studies was assessed using the I^2^ statistic. The random effects model and fixed effects model was used to pool the risk ratio (RR) with a corresponding 95% confidence interval (CI). A subgroup analysis was performed based on different methods of ultrasonic measurement and different endometrial compaction rates (ECR). Stata 17.0 software was used for meta-analysis. Pregnancy outcomes, which included clinical pregnancy rate, ongoing pregnancy rate, live birth rate, and spontaneous abortion rate, were evaluated.

**Result(s):**

In this study, 18 cohort studies were included, involving 16,164 embryo transfer cycles. Pooled results indicated that there was no significant difference between the endometrial compaction group and the non-compaction group in terms of clinical pregnancy rate (RR [95% CI]=0.98 [0.90,1.08]; I^2 = ^69.76%), ongoing pregnancy rate (RR [95% CI]=1.18 [0.95,1.47]; I^2 = ^78.77%), live birth rate (RR [95% CI]= 0.97 [0.92,1.02]; I^2 = ^0.00%) or spontaneous abortion rate (RR [95% CI]= 1.07[0.97,1.26]; I^2 = ^0.00%). According to the subgroup analysis of ultrasonic measurement methods, in the transvaginal ultrasound (TVUS) combined with abdominal ultrasonography (AUS) cycles of the endometrial compaction group, the rate of ongoing pregnancy (RR [95% CI] = 1.69 [1.26, 2.26]; I^2 = ^29.27%) and live birth (RR [95% CI] = 1.27 [1.00,1.61]; I^2 = ^62.28%) was significantly higher than that of the non-compaction group. Additionally, subgroup analysis based on ECR revealed a significantly higher rate of ongoing pregnancy when ECR ≥ 15% (RR [95% CI] = 1.99 [1.61, 2.47]; I^2 = ^0.00%).

**Conclusion:**

Endometrial compaction has no adverse effect on clinical pregnancy rate, ongoing pregnancy rate, live birth rate, or spontaneous abortion rate. A possible explanation for the contradictory findings of previous studies lies in the method by which the EMT is measured.

**Systematic review registration:**

https://www.crd.york.ac.uk/prospero/display_record.php?ID=CRD42023430511, identifier CRD42023430511.

## Introduction

Infertility has become a major public health concern worldwide. Current evidence indicates a 15.5% prevalence of infertility, with 55.2% of couples seeking medical care (1). Medical advances have made assisted reproduction a viable option for infertile couples worldwide. Statistically, ongoing pregnancy rates following IVF vary between 8.6% and 46.2% per cycle (2), depending on embryo quality, endometrial receptivity (ER), and embryo-endometrial communication (3). Preimplantation genetic testing (PGT) has become increasingly common in recent years, which to some extent has improved the quality of implanted embryos and reduced the effects of aneuploid embryos on IVF. Yet, its positive predictive value is not higher than 50-60% (4). Hence, ER evaluation is essential before embryo transfer (ET).

Good ER enables the endometrium to provide an optimal environment for embryo development and placenta formation. EMT is the most widely recognized ER marker (5). EMT measurement by ultrasound is also routinely performed in clinical practice as a non-invasive method of ER evaluation. For ER, a “window of implantation” occurs when the endometrium is at its best to support trophoblast-endometrial interactions, which is thought to occur around days 22-24 of an ideal 28-day cycle (6). In previous studies, EMT has been studied on the day human chorionic gonadotropin (hCG) is triggered in fresh ET cycles, as well as on the day when estrogen is discontinued or progesterone is begun in frozen-thawed embryo transfer (FET) cycles (7, 8).

Nevertheless, there is still controversy regarding the predictiveness of EMT on pregnancy outcomes (9). In many cycles, ET has been postponed due to inadequate EMT. A recent meta-analysis demonstrated that a thin endometrium is associated with poor live birth rates (LBR) (10). LBRs are decreased when EMT is less than 8 mm on the day of hCG in a fresh ET cycle or when EMT is less than 7 mm on the day of progestogen initiation in a FET cycle (8). However, the study by Ata et al. (11) evaluated the effect of EMT and LBR after the transfer of 959 single euploid blastocysts without EMT cutoff. They could not find a threshold below which LBR decreased, and concluded that an endometrium of 3-4 mm has a similar LBR to a thicker endometrium.

Though the above studies have mainly examined EMT, which is measured before hCG administration or progesterone administration, few have looked at how EMT changes during the end of the follicular phase or the early luteal phase influence pregnancy outcomes. Haas et al. (12) reported that endometrial compaction (thinning of the EMT during the end of the follicular phase or early luteal phase) may lead to more favorable pregnancy outcomes, but subsequent studies have made conflicting findings (13, 14). In the present study, we sought to review the evidence from observational studies to explore whether endometrial compaction is predictive of pregnancy outcomes and to inform reproductive clinicians in their assessment of ER.

## Materials and methods

The systematic review and meta-analysis was conducted according to the Preferred Reporting Items for Systematic Reviews and Meta-Analyses (PRISMA) checklist (15).

### Search strategy

PubMed, Cochrane, Embase, and Web of Science electronic databases were searched for studies published in English up to March 2023, using Medical Subject Headings [MeSH] and keywords related to the study, no restriction on the year of publication. The database’s specific strategy was (‘Embryo Transfer’ OR ‘Embryo Transfers’ OR ‘Blastocyst Transfer’ OR ‘Blastocyst Transfers’ OR ‘Fertilizations in Vitro’ OR ‘IVF’ OR ‘*In Vitro* Fertilization’ OR ‘*In Vitro* Fertilizations’ OR ‘ICSI’ OR ‘Intracytoplasmic Sperm Injection’ OR ‘Intracytoplasmic Sperm Injections’) AND (‘Endometrial compaction’ OR ‘Endometrial thickness change’ OR ‘change, endometrial thickness’ OR ‘Endometrial thickness decreased’ OR ‘Endometrial thickness compacted’) AND (‘Pregnancy Outcome’ OR ‘Pregnancy Outcomes’ OR ‘Clinical outcomes’ OR ‘Live birth’ OR ‘Clinical pregnancy’ OR ‘Ongoing pregnancy’). Additionally, references of all included articles were screened, and literature retrieval was finalized. The entire retrieved literature was screened by two independent reviewers, and the included literature was collated using EndNote X9.3.3. Any articles with uncertainties were resolved through discussion and, if necessary, group discussion with a third investigator to reach a final consensus.

### Inclusion/exclusion criteria

Studies that met the following criteria were considered eligible for systematic review: (1) The study was an observational cohort study; (2) The study population was all women who underwent IVF; (3) Report on the relationship between EMT changes and pregnancy outcomes; (4) Studies had at least one of the following outcomes: clinical pregnancy, ongoing pregnancy, live birth, and spontaneous abortion; (5) Published in English.

Studies with the following conditions will be excluded: (1) No clinical outcome or no available data; (2) Overview, conference abstracts, case reports, and case series; (3) Articles without complete research strategy.

### Outcome measures

Clinical pregnancy is defined as one or more gestational sacs seen in the uterine cavity detected by ultrasound. Ongoing pregnancy is defined as transvaginal ultrasound showing fetal heart activity at 12 weeks of gestation or later. Live birth is defined as surviving infants delivered at ≥ 24 weeks of gestation. Spontaneous abortion is considered to be the pregnancy loss of one or more gestational sacs previously observed before 24-week gestation.

### Data extraction

According to the PRISMA guidelines, the following data were extracted independently by two authors from each eligible study: first author’s surname, publication year, country, study duration, study design, number of cycles, endometrial preparation protocol, endometrial measurement method, number of embryos transferred, embryo development stage, and pregnancy outcomes. Data from different subgroups within the same study were also extracted for possible synthesis. Disagreements between the two reviewers were resolved in the same manner as described above.

### Quality assessment

The quality of the included cohort studies was assessed independently by two reviewers using the Newcastle-Ottawa Scale (NOS), which assigns a maximum of nine points to each study based on three broad dimensions: selection (4 points), comparability (2 points), and outcome (3 points). Scores ranged from 0 to 9 points, with studies ≥ 7 points being considered high quality, 4-6 points indicating moderate quality, and < 4 points indicating low quality. Disagreements between the two reviewers were resolved in the same manner as described above.

### Statistical analysis

Pregnancy outcomes were counted as dichotomous variables and expressed as RR with a 95% CI. The degree of heterogeneity was quantified by I^2^ statistics, when I^2 = ^0%, considered no heterogeneity between studies, when I^2^ < 25%, considered mild heterogeneity between studies, when 25% ≤ I^2^ < 50%, considered moderate heterogeneity between studies, when I^2^ ≥ 50%, considered high heterogeneity between studies (16). According to heterogeneity, the results were calculated using a random effects model (Der Simonian-Laird) or fixed-effects model (Mantel-Haenszel). All statistical analyses were performed using Stata 17.0.

## Results

### Literature search and study features

**Figure 1:**
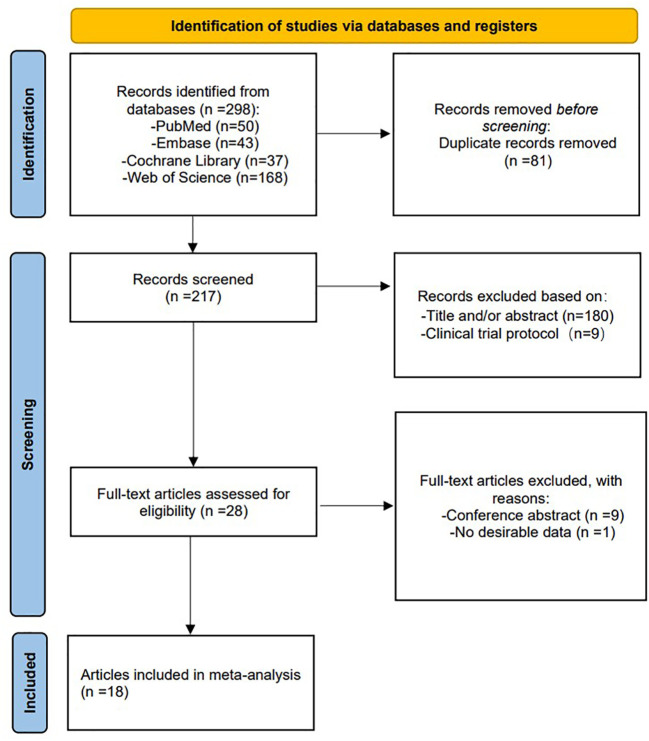
PRISMA flow diagram of search and selection strategy.

In total, 298 studies were retrieved and reviewed. 217 studies were retained after removing duplicates, with each title and abstract being evaluated by two reviewers. Subsequently, 28 full-text articles were screened for a full review, and 10 articles were excluded for the reasons outlined in the flowchart, leaving 18 studies (12–14, 17–31) that met the inclusion criteria. The PRISMA scheme for searching and selecting literature is shown in **Figure 1**.

**Table 1** shows the basic characteristics of the 18 studies, including first author’s surname, publication year, country, study duration, study design, number of cycles, endometrial preparation protocol, endometrial measurement method, number of embryos transferred, embryo development stage, outcome indicators, and quality score. Seven prospective studies and 11 retrospective studies were eligible for meta-analysis. Among the 18 included studies, 3 examined fresh ET, 15 examined FET. A total of 16,164 cycles were included, including 3022 fresh ET cycles, and 13,142 FET cycles (including 6481 hormone replacement therapy [HRT] cycles and 6661 natural cycles [NC] or modified natural cycles [mNC]).

**Table 1:** Main characteristics.

Study	Year	Country	Duration	Design	No. of cycles	Treatment	IVF status	Embryo stage	No. of embryos transferred	Endometrial preparation	Measurement	Outcomes	NOS score
Bu	2019	China	2015.4-2019.3	Prospective cohort study	3091	IVF/ISCI	Frozen	Blastocyst stage	1	HRT/NC	TVUS	CPR	8
Haas	2019	Canada	2017.3-2018.8	Retrospective cohort study	271	IVF/PGT-A	Frozen	Blastocyst stage	1	HRT	TVUS+AUS	OPR	7
Zilberberg	2020	Canada	2016.6-2018.10	Retrospective cohort study	225	PGT-A	Frozen	Blastocyst stage	1	HRT	TVUS+AUS	OPR	6
Ye	2020	China	2010.1-2015.6	Retrospective cohort study	4465	IVF/ISCI	Frozen	Cleavage stage	1-2	HRT/NC	TVUS	CPR/LBR/SABR	9
Huang	2020	China	2011.1-2015.6	Retrospective cohort study	2768	IVF/ISCI	Frozen	Cleavage stage/blastocyst stage	1-2	mNC	TVUS	CPR/OPR/LBR/SAB	9
Riestenberg	2021	USA	2018.1-2018.12	Prospective cohort study	259	PGT-A	Frozen	Blastocyst stage	1	HRT	TVUS	CPR/LBR/SABR	8
Al Jarrah	2021	Iraq	2019.10-2020.4	Prospective cohort study	60	ICSI	Frozen	Cleavage stage	2-3	HRT	TVUS	CPR/OPR/SABR	7
Huang	2021	China	2003.1-2012.12	Retrospective cohort study	2620	IVF/ISCI	Fresh	Cleavage stage/blastocyst stage	1-3	–	TVUS	CPR/OPR/LBR/SAB	9
Yaprak	2021	Turkey	2013.5-2019.10	Retrospective cohort study	283	ISCI	Frozen	Cleavage stage/blastocyst stage	1-2	HRT	TVUS+AUS	CPR/LBR/SABR	8
Jin (a)	2021	China	2014.1-2019.12	Retrospective cohort study	508	PGT-SR/PGT-M	Frozen	Blastocyst stage	1	HRT	TVUS	CPR/LBR/SABR	8
Jin (b)	2021	China	2014.1-2019.12	Retrospective cohort study	219	PGT-SR/PGT-M	Frozen	Blastocyst stage	1	NC	TVUS	CPR/LBR/SABR	8
Kaye	2021	USA	2018.5-2019.12	Retrospective cohort study	232	IVF/PGT-A	Frozen	Blastocyst stage	1	HRT	TVUS	CPR/OPR/SABR	8
Shah	2022	USA	2020.9-2021.4	Prospective cohort study	186	PGT-A	Frozen	Blastocyst stage	1	HRT/mNC	TVUS+AUS	CPR/LBR	7
Youngster	2022	Israel	2019.8-2021.7	Prospective cohort study	71	IVF	Frozen	Cleavage stage/blastocyst stage	1-2	NC	TVUS	CPR/OPR/SABR	8
Olgan	2022	Turkey	2020.12-2021.4	Prospective cohort study	204	IVF/ISCI	Frozen	Blastocyst stage	1-2	HRT	TVUS/TVUS+AUS	CPR/OPR/SABR	8
Lam	2022	China	2005.6-2006.8	Retrospective cohort study	268	IVF/ISCI	Fresh	Cleavage stage	1-3	–	3D TVUS	LBR	9
Gursu	2022	Turkey	2017.1-2019.12	Retrospective cohort study	134	ICSI	Fresh	Blastocyst stage	1-2	–	AUS	CPR/LBR	8
Jafarabadi	2023	Iran	2020.3-2021.3	Prospective cohort study	300	–	Frozen	Cleavage stage	1-2	HRT	TVUS	CPR/OPR/SABR	8

HRT, hormone replacement therapy; NC, natural cycles; mNC, modified natural cycles; TVUS, transvaginal ultrasonography; AUS, abdominal ultrasonography; CPR, clinical pregnancy rate; OPR, ongoing pregnancy rate; LBR, live birth rate; SABR, spontaneous abortion rate.

In five studies of FET-HRT (13, 14, 22, 23, 31) and one study with fresh oocyte donation (26), ECR was defined as the rate of change in EMT between the day of progesterone administration and the day of ET. In 7 other FET-HRT studies (12, 18–20, 24, 25, 28), ECR was defined as the rate of change at which EMT changed from the end of estrogen-only phase to the ET or the day before the ET. In 2 fresh ETs (21, 27) and 2 FET-mNC (17, 29) studies, ECR refers to the rate at which EMT changes from hCG triggered to ET. Youngster et al. (30) described ECR as the rate of change from the day of ovulation to the day of ET.

### Meta-analysis of clinical pregnancy rate

**Figure 2:**
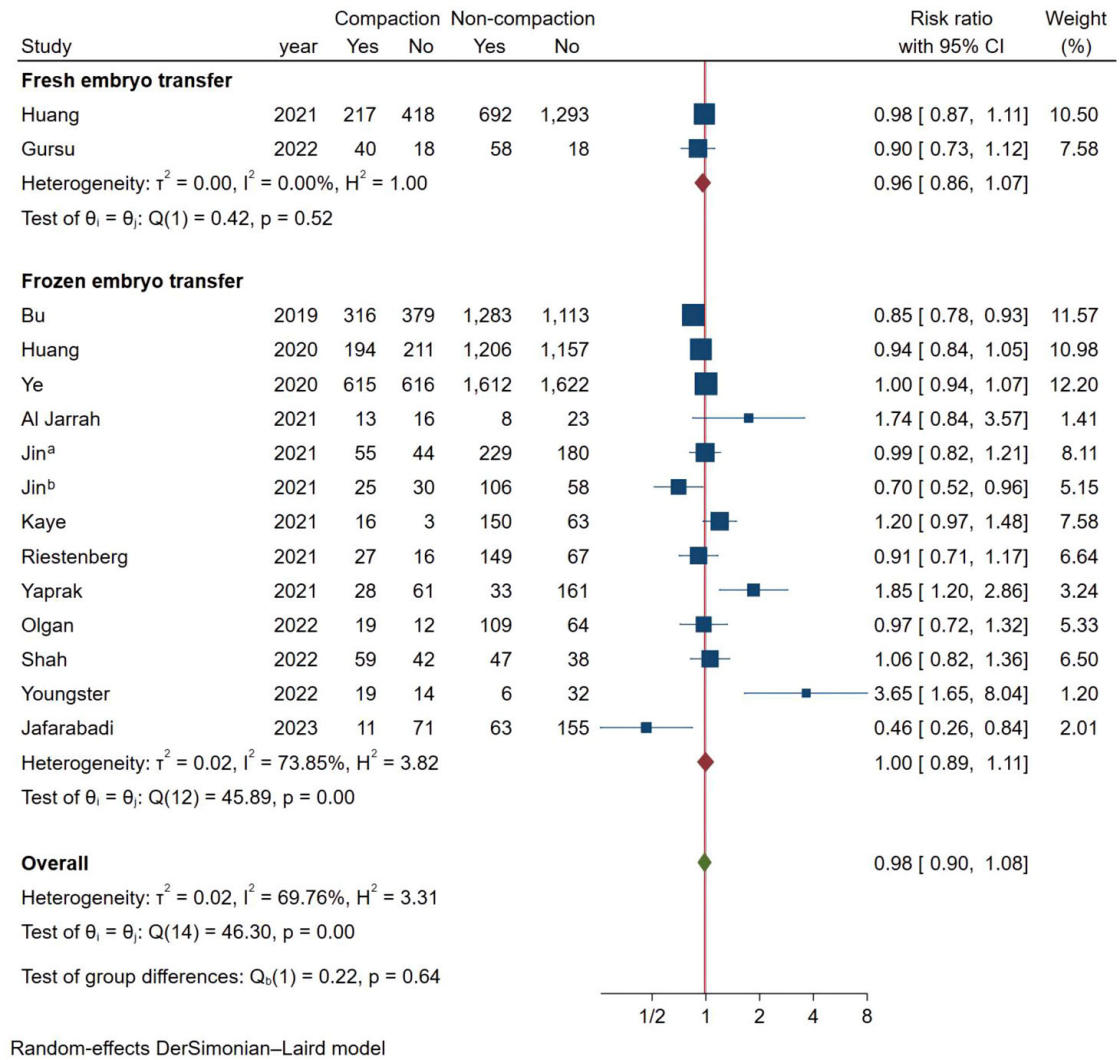
Forest plot of clinical pregnancy.

As shown in **Figure 2**, a total of 15 studies documented CPR, and the pooled results indicated that endometrial compaction was not associated with CPR (RR [95% CI] = 0.98 [0.90,1.08]; I^2 = ^69.76%), but there was high heterogeneity across the studies. Subgroup analysis revealed no statistically significant difference in CPR between endometrial compaction and non-compaction groups in the Fresh ET subgroup (RR [95% CI] = 0.96 [0.86,1.07]; I^2 = ^0.00%) or the FET subgroup (RR [95% CI] = 1.00 [0.89,1.11]; I^2 = ^73.85%). Moreover, we also performed subgroup analysis according to different preparation protocols, study design, measurement methods, and ECR. As shown in **Table 2**, there was no correlation between endometrial compaction and CPR.

**Table 2:** Subgroup analysis of clinical pregnancy.

Subgroups		RR	95% CI
**Frozen embryo transfer**	HRT	1.01	0.87,1.16
	NC	0.93	0.80,1.09
**Design**	Prospective cohort	1.00	0.79,1.25
	Retrospective cohort	0.99	0.91,1.09
**Change ratio**	0%	0.98	0.86,1.10
	5%	0.97	0.78,1.21
	10%	0.98	0.83,1.16
	15%	1.14	0.72,1.81
	20%	0.79	0.57,1.09
**Measurement**	AUS	0.90	0.73,1.12
	TVUS	0.96	0.87,1.06
	TVUS+AUS	1.36	0.79,2.34

HRT, hormone replacement therapy; NC, natural cycles; mNC, modified natural cycles; TVUS, transvaginal ultrasonography; AUS, abdominal ultrasonography; RR, risk ratio; CI, confidence interval.

### Meta-analysis of ongoing pregnancy rate

**Figure 3:**
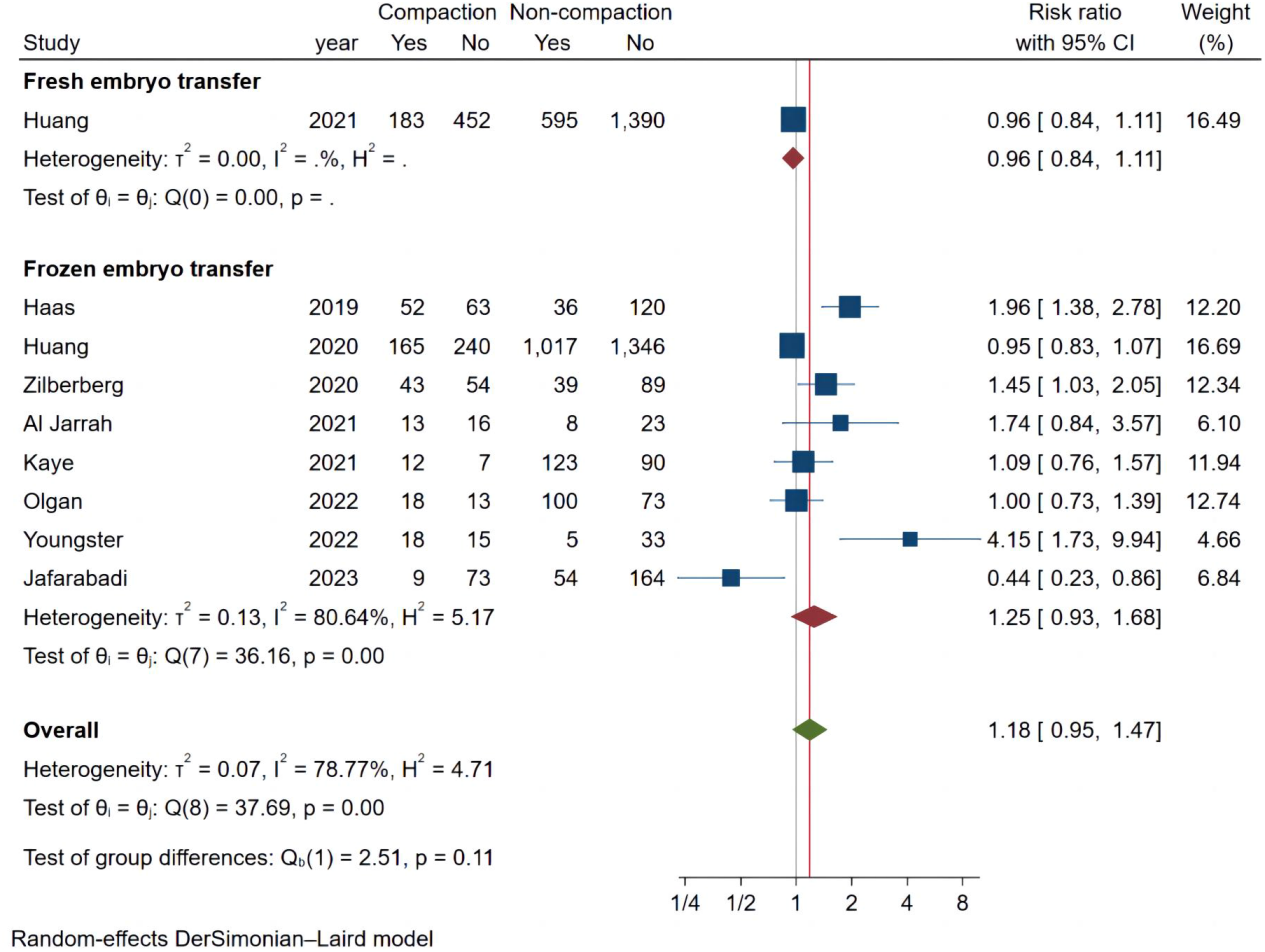
Forest plot of ongoing pregnancy.

We analyzed 9 studies that met the requirements and the combined results are shown in **Figure 3**, where endometrial compaction was not associated with the OPR (RR [95% CI] = 1.18 [0.95,1.47]; I^2 = ^78.77%). There was high heterogeneity among studies, so we also performed a subgroup analysis with only one study in the Fresh ET group (RR [95% CI] = 0.96[0.84,1.11]; I^2 = ^0.00%) and 8 studies in the FET group (RR [95% CI] = 1.25 [0.93,1.68]; I^2 = ^80.64%), all of which showed no correlation between endometrial compaction and OPR.

**Figure 4:**
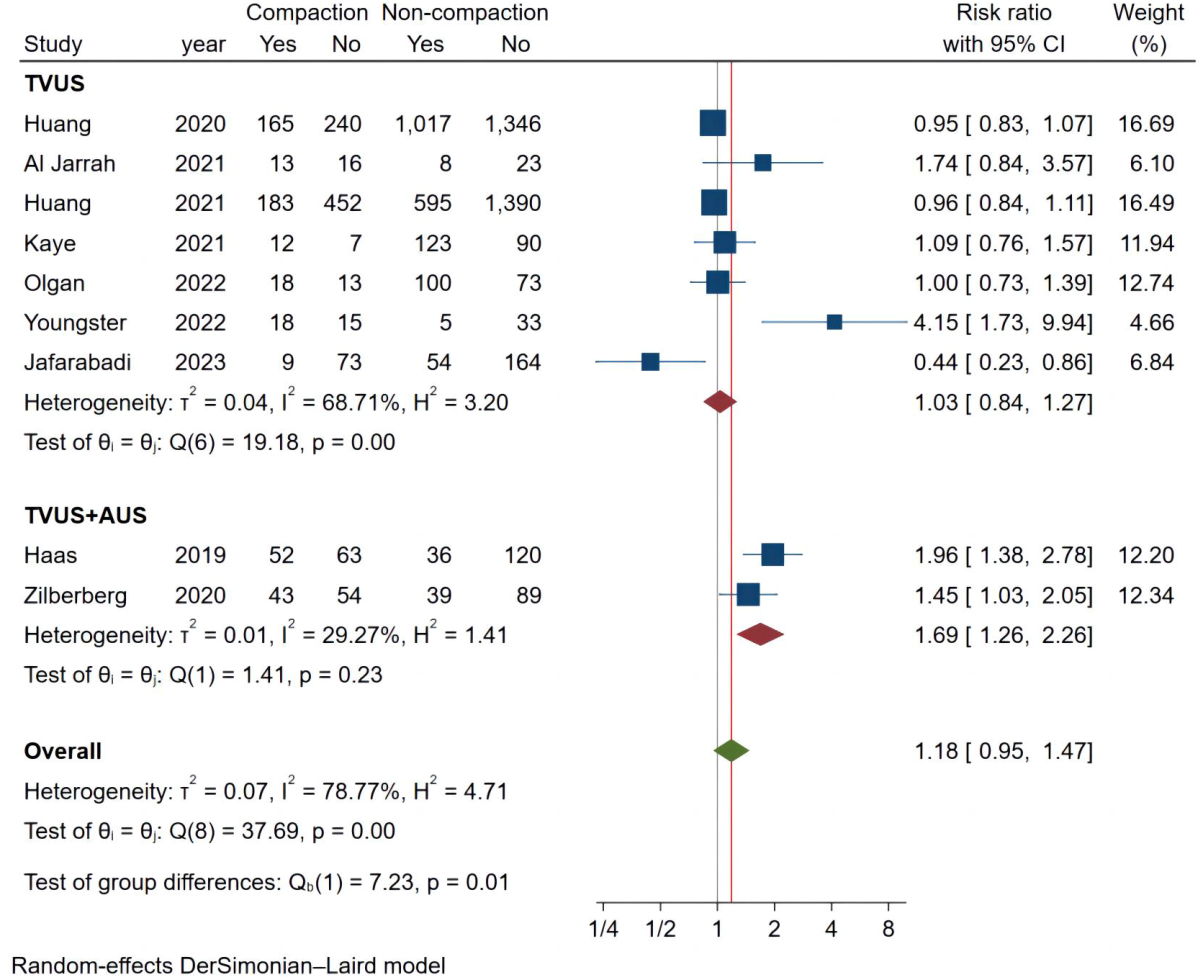
Subgroup analysis of measurement methods in ongoing pregnancy.

**Figure 5:**
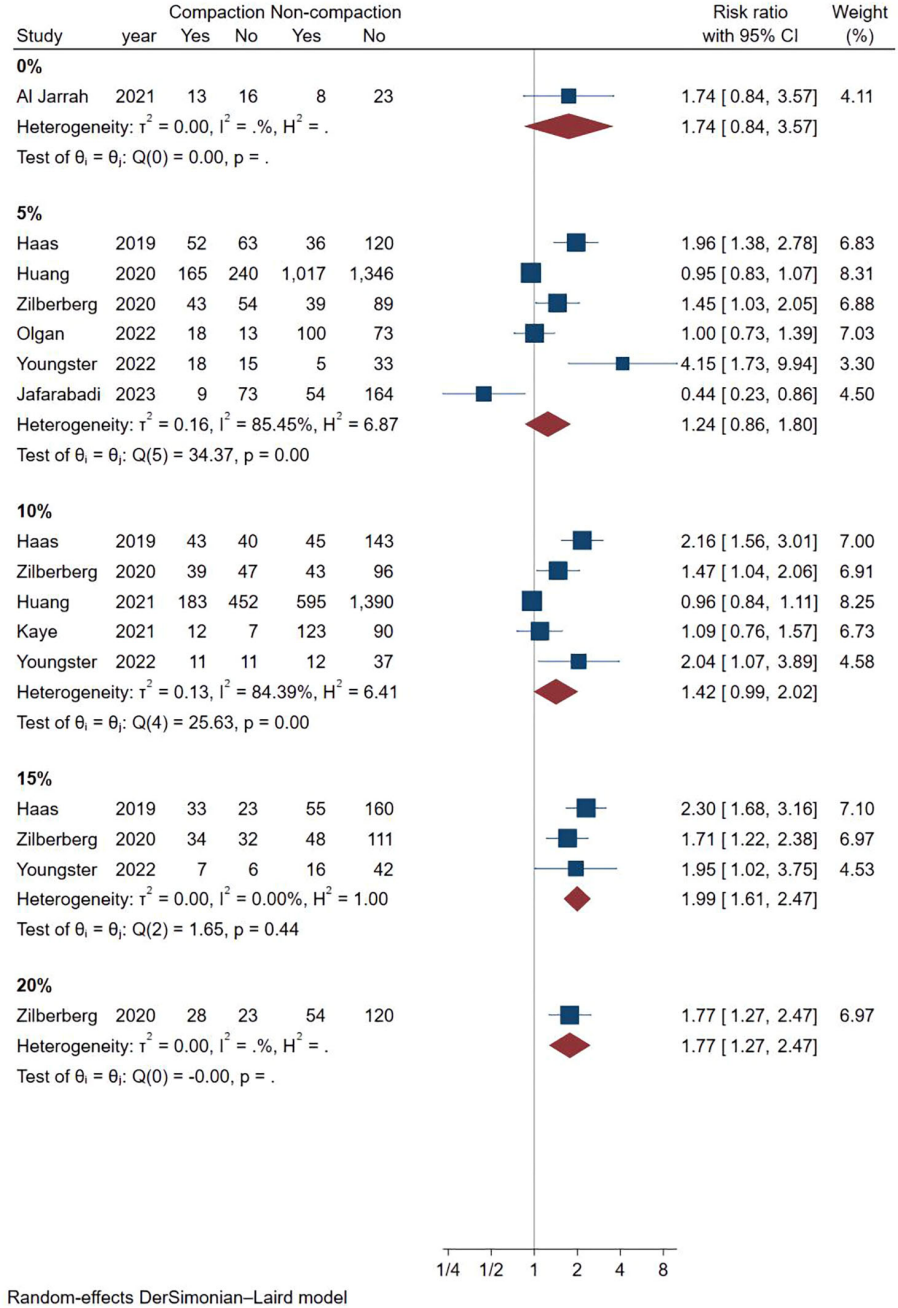
Subgroup analysis of compaction rate in ongoing pregnancy.

Considering the high heterogeneity between studies, we conducted more subgroup analyses. In a subgroup analysis of different ultrasound measurement methods, the OPR of the endometrial compaction group in the ET cycle was significantly higher than that of the non-compaction group (RR [95% CI]=1.69 [1.26, 2.26]; I^2 = ^29.27%), based on the combination of the first TVUS measurement and the second AUS measurement of EMT (see **Figure 4**). According to a subgroup analysis of different ECRs, the OPR was higher in the endometrial compaction group than in the non-compaction group (RR [95% CI] = 1.99 [1.61,2.47]; I^2 = ^0.00%). In the other subgroups, similar results were not observed, as shown in **Figure 5**. No statistical differences were found between the endometrial compaction and non-compaction groups in the subgroup analysis of other influencing factors, as shown in **Table 3**.

**Table 3:** Subgroup analysis of ongoing pregnancy.

Subgroups		RR	95% CI
**Frozen embryo transfer**	HRT	1.19	0.85,1.68
	NC	1.85	0.44,7.84
**Design**	Prospective cohort	1.27	0.60,2.68
	Retrospective cohort	1.18	0.94,1.47
**Change ratio**	0%	1.74	0.84,3.57
	5%	1.24	0.86,1.80
	10%	1.42	0.99,2.02
	15%	1.99	1.61,2.47
	20%	1.77	1.27,2.47
**Measurement**	TVUS	1.03	0.84,1.27
	TVUS+AUS	1.69	1.26,2.26

HRT, hormone replacement therapy; NC, natural cycles; TVUS, transvaginal ultrasonography; AUS, abdominal ultrasonography; RR, risk ratio; CI, confidence interval.

### Meta-analysis of live birth

**Figure 6:**
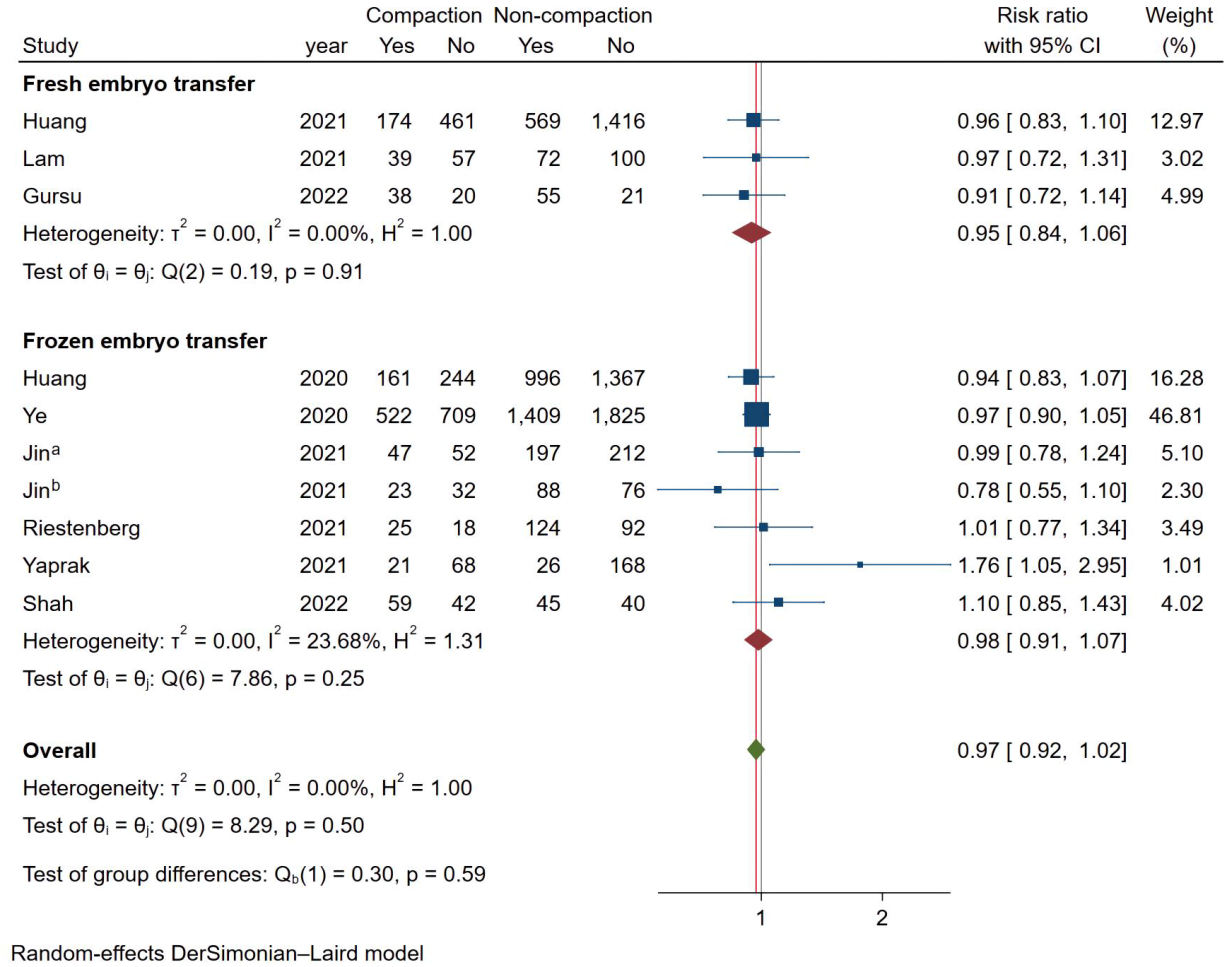
Forest plot of live birth.

As shown in **Figure 6**, a total of 10 studies reported LBR using a fixed effects model (Mantel-Haenszel), and the combined results showed no statistically significant association between endometrial compaction and LBRs (RR [95% CI] = 0.97 [0.92,1.02]; I^2 = ^0.00%). In subgroup analysis, the Fresh ET group (RR [95% CI] = 0.95 [0.85,1.07]; I^2 = ^0.00%) and FET group (RR [95% CI] = 0.98 [0.92,1.04]; I^2 = ^23.68%) showed no significant difference between the endometrial compaction and non-compaction.

**Figure 7:**
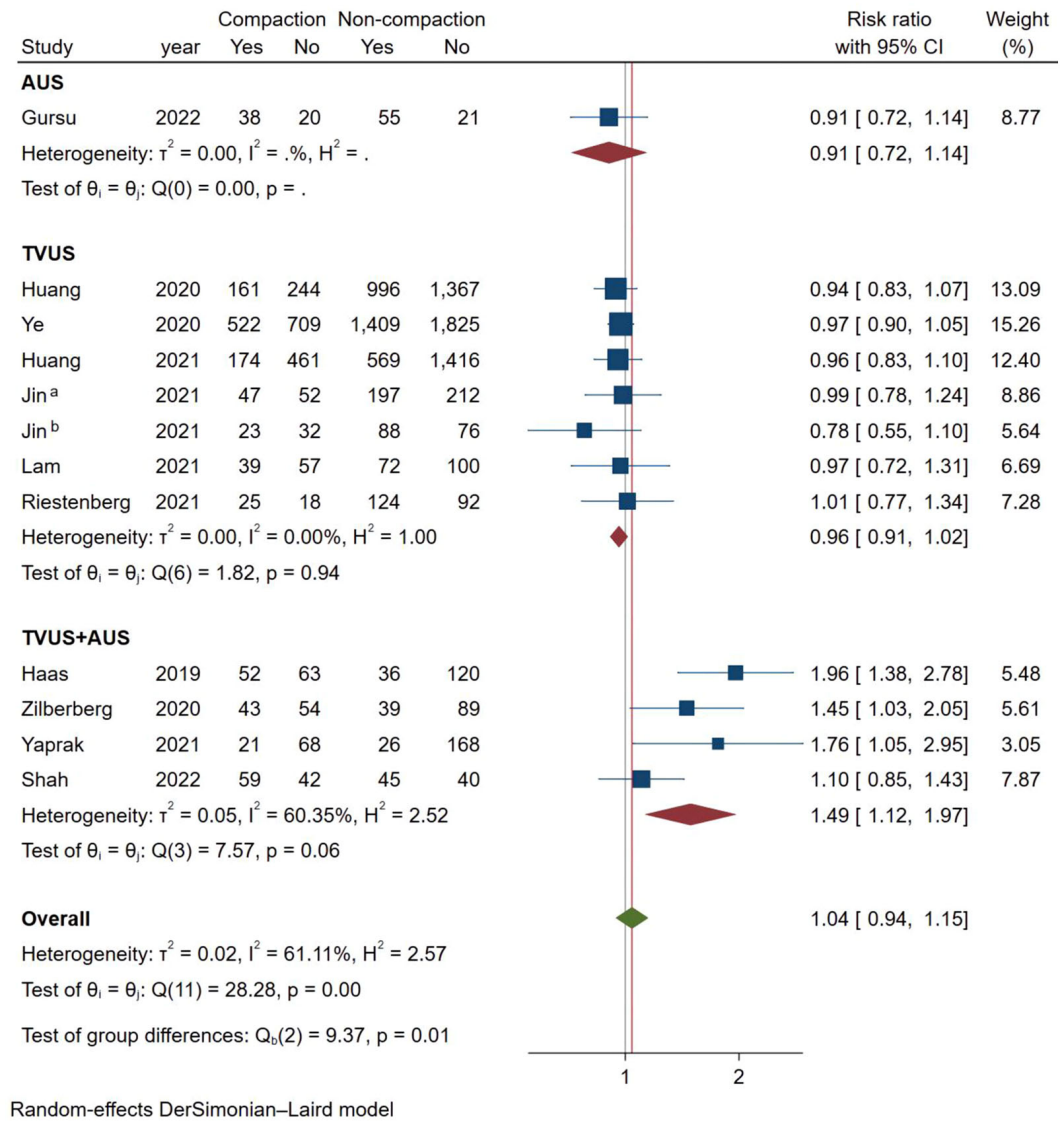
Subgroup analysis of measurement methods in live birth.

In the subgroup analysis of TVUS + AUS, the endometrial compaction group showed a slight improvement in LBR (RR [95% CI]=1.27 [1.00,1.61]; I^2 = ^62.28%). However, as shown in **Figure 7**, no significant statistical difference in LBR was detected when TVUS was used for both measurements prior to ET (RR [95% CI] = 0.96 [0.91,1.02]; I^2 = ^0.00%). As shown in **Table 4**, there were no significant differences in pooled RRs of LBR after conducting other subgroups analyses.

**Table 4:** Subgroup analysis of live birth.

Subgroups		RR	95% CI
**Frozen embryo transfer**	HRT	1.02	0.93,1.11
	NC	0.95	0.87,1.03
**Design**	Prospective cohort	1.06	0.88,1.28
	Retrospective cohort	0.97	0.91,1.02
**Change ratio**	0%	0.98	0.92,1.05
	5%	1.00	0.91,1.10
	10%	0.97	0.90,1.04
	15%	0.95	0.79,1.16
	20%	1.00	0.86,1.16
**Measurement**	TVUS	0.91	0.72,1.14
	TVUS+AUS	0.96	0.91,1.02

HRT, hormone replacement therapy; NC, natural cycles; TVUS, transvaginal ultrasonography; AUS, abdominal ultrasonography; RR, risk ratio; CI, confidence interval.

### Meta-analysis of spontaneous abortion

**Figure 8:**
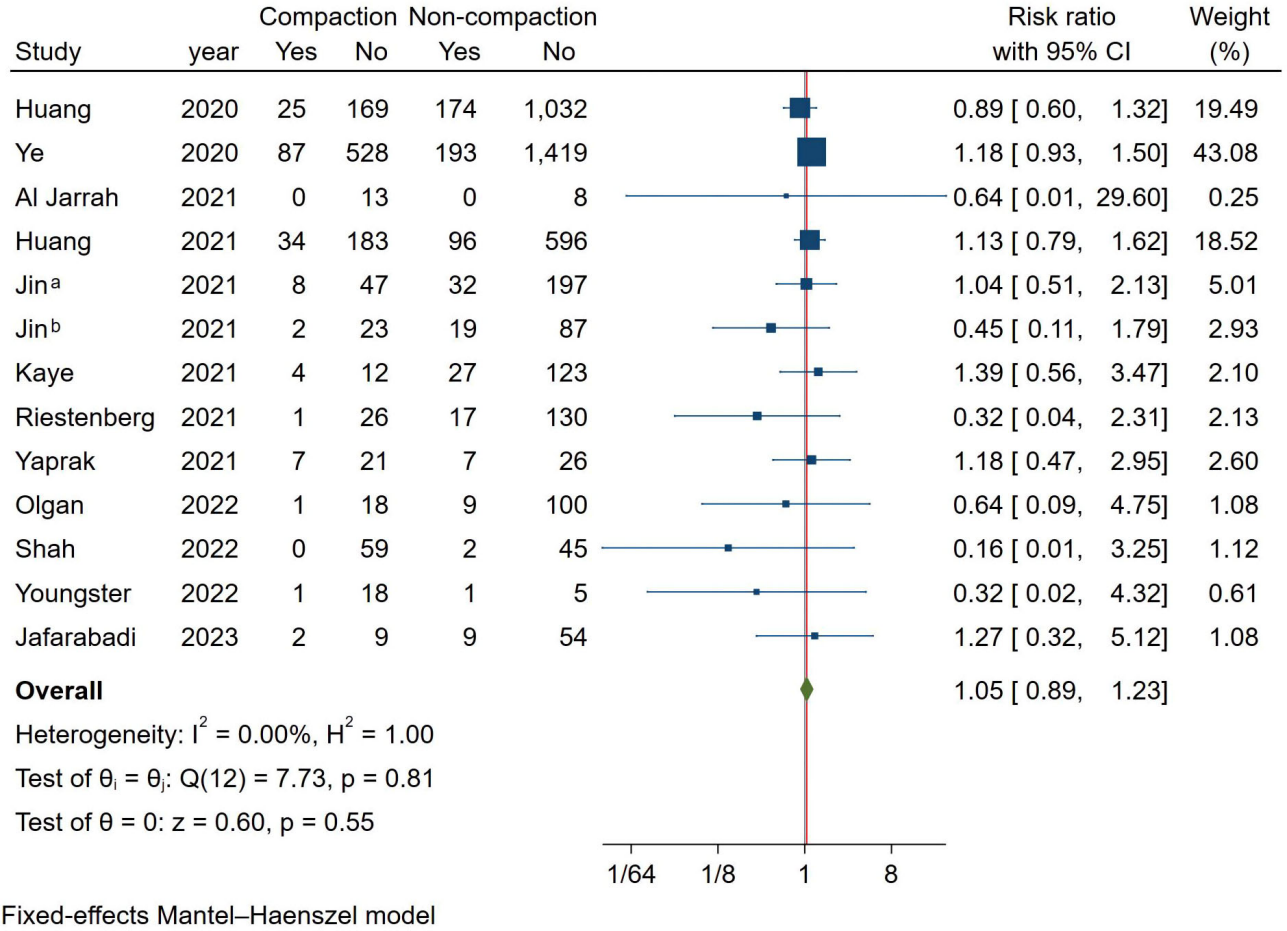
Forest plot of spontaneous abortion.

As shown in **Figure 8**, we analyzed all studies that met the criteria. Because of the low heterogeneity between studies, a fixed-effect model with the Mantel-Haenszel method was used. Combined results showed that endometrial compaction was not associated with spontaneous abortion (RR [95% CI] = 1.05 [0.89,1.23]; I^2 = ^0.00%).

### Publication bias

The funnel plot for each outcome was visually symmetric (see **Supplementary Appendix 3-1**, **3-2**, **3-3**, **3-4**). Furthermore, the regression-based Egger test did not show statistical significance (P = 0.0512, 0.120, 0.4295 and 0.0727 for clinical pregnancy, ongoing pregnancy, live birth and spontaneous abortion, respectively), suggesting that there was no significant publication bias in the studies that were included.

## Discussion

EMT is currently used widely in the field of IVF as a clinical predictor of pregnancy outcomes. Most previous studies focused on EMT’s effect on pregnancy outcomes in IVF. Generally, thinner endometrium is associated with poorer pregnancy outcomes (2, 32). In the last decade, only a few studies have focused on the effect of endometrial compaction on pregnancy outcomes, and the results are contradictory. Thus, we designed this systematic review to evaluate the impact of endometrial compaction on IVF outcomes. CPR, OPR, LBR, and spontaneous abortion rates were not significantly affected by endometrial compaction. However, a subgroup analysis showed that endometrial compaction may be associated with an increase in OPR and LBR when TVUS was used for the first measurement of EMT and AUS was used for the second measurement of EMT. Meanwhile, when ECR ≥ 15%, LBR also appeared to be better, despite significant heterogeneity between studies.

A total of 7 prospective studies and 11 retrospective studies were included. Of these, 11 studies (17, 18, 20–22, 24, 26–29, 31) did not find a statistically significant association between endometrial compaction and pregnancy outcomes, whereas in 5 studies (12, 19, 23, 25, 30) better pregnancy outcomes were reported. In contrast, Bu et al. (14) and Jin et al. (13) concluded that an increased endometrium after progesterone administration was associated with better pregnancy outcomes. In the present meta-analysis, combined results showed that endometrial compaction was not associated with CPR, OPR, LBR, or spontaneous abortion rate.

Because of the heterogeneity among studies, we performed subgroup analyses. The subgroup analysis according to the ultrasound measurement method indicated that using AUS at the time of ET resulted in higher OPR and LBR in the endometrial compaction group than in the non-compaction group. These findings are consistent with those reported by Haas (12), Zilberberg (19), and Yaprak (25). While continuous EMT monitoring using TVUS did not reveal a correlation between endometrial compaction and pregnancy outcome, in the subgroup analysis. It is generally accepted that EMT measured by TVUS are more accurate than those measured by AUS. According to our hypothesis, AUS measurement results in a thinner EMT as well as a higher incidence of endometrial compaction as a result of pressure placed on the abdomen while AUS measurements are taken. The study of Olgan et al. (28) compared two different measurements and the conclusions obtained were consistent with our conjecture. We believe that the data measured by sequential TVUS are more reliable than those measured by TVUS and AUS. Hence, TVUS may be a good choice for future researchers who wish to reduce measurement errors.

In this study, we also performed subgroup analysis according to different ECRs. Our study showed that endometrial compaction resulted in a higher OPR when 15% was used as a cut-off value for ECR. However, two of the three studies included combined transvaginal and transabdominal measurements. As a consequence of the small number of included studies, it is not possible to exclude the effect of measurement methods on study outcomes.

In the FET-HRT analysis, endometrial compaction did not significantly affect OPR and LBR. Nonetheless, this compares with Oliveira et al. ‘s findings (33), which found that endometrial compaction during FET-HRT cycles benefited OPR and LBR. In our study, we analyzed OPR and LBR separately and included data from more recent studies, which may explain the differences. Therefore, more well-designed clinical trials are needed to validate an innovative concept before it can be introduced into clinical practice.

Additionally, the researchers observed the changes in endometrium during the natural cycle and found that the thickness and volume increased rapidly during the follicular phase and decreased slightly after ovulation (34, 35). In the follicular phase, estrogen accelerates the proliferation and growth of endometrial glands and vessels, which exhibit a typical trilinear ultrasound sign. After ovulation, the endometrium becomes more curved and vascularized by progesterone, and the EMT becomes thinner or unchanged, which appears hyperechoic on ultrasound. Researchers (12, 19, 20, 23, 30, 36) reported that the degree of endometrial compaction can indicate the degree of endometrial response to progesterone and contribute to an assessment of ER. However, Olgan et al. (28) and Youngster et al. (30) demonstrated that progesterone levels and estrogen to progesterone ratio were not associated with endometrial compaction. Thus, further studies may identify factors affecting endometrial compaction and how endometrial compaction improves pregnancy outcomes from the perspective of progesterone resistance and progesterone receptor expression.

There are some limitations to this meta-analysis. First of all, most of the included literature is retrospective cohort studies. Secondly, there are differences in embryo culture methods, ET staging, EMT measurement methods, ECR thresholds, and clinical outcome evaluation. This results in significant heterogeneity between individual studies. Further, only three studies on fresh ET were included, including one on the oocyte donation cycle. Insufficient studies were included to obtain reliable results.

## Conclusion

In summary, the present evidence suggests that endometrial compaction is not sufficient for predicting pregnancy outcomes. Also, the choice of endometrial measurement methods is a key factor influencing endometrial compaction assessment. Besides reducing unnecessary cycle cancellations, this finding may also provide an instructive basis for future studies. To confirm this finding, more well-designed, large-scale prospective studies should be conducted.

## Data availability statement

The original contributions presented in the study are included in the article/supplementary material. Further inquiries can be directed to the corresponding author.

## Author contributions

X-TC: Data curation, Formal Analysis, Writing – original draft. Z-GS: Conceptualization, Funding acquisition, Methodology, Writing – review & editing, Data curation. J-YS: Conceptualization, Software, Supervision, Writing – review & editing.

## Supplementary material

Supplementary Appendix 1 Search strategy

Supplementary Appendix 2 Newcastle-Ottawa Scale

Supplementary Appendix 3-1 Funnel plot of clinical pregnancy.

Supplementary Appendix 3-2 Funnel plot of ongoing pregnancy.

Supplementary Appendix 3-3 Funnel plot of live birth.

Supplementary Appendix 3-4 Funnel plot of spontaneous abortion.
