# Supplementary material for: Does endometrial compaction before embryo transfer affect pregnancy outcomes? a systematic review and meta-analysis
Source: Front Endocrinol (Lausanne). 2023 Nov 14;14:1264608. doi: 10.3389/fendo.2023.1264608 (PMC10682779; doi:10.3389/fendo.2023.1264608)
Supplement: Supplementary Appendix 1 — Search strategy [file Table_1.docx]

2023-03-06

PUBMED：50

| #1 |  | Search:**(((((Embryo Transfer[MeSH Terms]) OR (Embryo Transfers[Title/Abstract])) OR (Transfer, Embryo[Title/Abstract])) OR (Transfers, Embryo[Title/Abstract])) OR (Blastocyst Transfer[Title/Abstract])) OR (Blastocyst Transfers[Title/Abstract])** | 19136 |
| --- | --- | --- | --- |
| #2 |  | Search: **(((Fertilizations in Vitro[MeSH Terms]) OR (In Vitro Fertilization[Title/Abstract])) OR (In Vitro Fertilizations[Title/Abstract])) OR (IVF[Title/Abstract])** | 55488 |
| #3 |  | Search: **(((((((Sperm Injections, Intracytoplasmic[MeSH Terms]) OR (ICSI[Title/Abstract])) OR (Injection, Intracytoplasmic Sperm[Title/Abstract])) OR (Injections, Intracytoplasmic Sperm[Title/Abstract])) OR (Intracytoplasmic Sperm Injection[Title/Abstract])) OR (Sperm Injection, Intracytoplasmic[Title/Abstract])) OR (Intracytoplasmic Sperm Injections[Title/Abstract])) OR (Injections, Sperm, Intracytoplasmic[Title/Abstract])** | 14484 |
| #4 |  | Search: **#1 OR #2 OR #3** | 63499 |
| #5 |  | Search: **((((Endometrial compaction[Title/Abstract]) OR (Endometrial thickness change[Title/Abstract])) OR (change, endometrial thickness[Title/Abstract])) OR (Endometrial thickness decreased[Title/Abstract])) OR (Endometrial thickness compacted[Title/Abstract])** | 549 |
| #6 |  | Search: **(((((((Pregnancy Outcome[MeSH Terms]) OR (Pregnancy Outcomes[Title/Abstract])) OR (Outcome, Pregnancy[Title/Abstract])) OR (Outcomes, Pregnancy[Title/Abstract])) OR (Clinical outcomes[Title/Abstract])) OR (Live birth[Title/Abstract])) OR (Clinical pregnancy[Title/Abstract])) OR (Ongoing pregnancy[Title/Abstract])** | 251395 |
| #7 |  | Search: **#4 AND #5 AND #6** | 50 |

COCHRANE LIBRARY：37

#1 MeSH descriptor: [Embryo Transfer] explode all trees

#2 (Embryo Transfers OR Transfer, Embryo OR Transfers, Embryo OR Blastocyst Transfer OR Blastocyst Transfers):ti,ab,kw (Word variations have been searched)

#3 MeSH descriptor: [Fertilization in Vitro] explode all trees

#4 (In Vitro Fertilization OR In Vitro Fertilizations OR Fertilizations in Vitro OR IVF):ti,ab,kw (Word variations have been searched)

#5 MeSH descriptor: [Sperm Injections, Intracytoplasmic] explode all trees

#6 (Injection, Intracytoplasmic Sperm OR Injections, Intracytoplasmic Sperm OR Intracytoplasmic Sperm Injection OR Sperm Injection, Intracytoplasmic OR Intracytoplasmic Sperm Injections OR ICSI OR Injections, Sperm, Intracytoplasmic):ti,ab,kw

#7 #1 OR #2 OR #3 OR #4 OR #5 OR #6

#8 (Endometrial compaction OR Endometrial thickness change OR Endometrial thickness decreased OR Endometrial thickness compacted):ti,ab,kw

#9 MeSH descriptor: [Pregnancy Outcome] explode all trees

#10 (Pregnancy Outcomes OR Outcome, Pregnancy OR Outcomes, Pregnancy OR Clinical outcomes OR Live birth OR Clinical pregnancy OR Ongoing pregnancy):ti,ab,kw

#11 #9 OR #10

#12 #7 AND #8 AND #11

EMBASE：43

#1 'embryo transfer':ti,ab,kw OR 'embryo transfers':ti,ab,kw OR 'transfer, embryo':ti,ab,kw OR 'transfers, embryo':ti,ab,kw OR 'blastocyst transfer':ti,ab,kw OR 'blastocyst transfers':ti,ab,kw

#2 'fertilizations in vitro':ti,ab,kw OR 'in vitro fertilization':ti,ab,kw OR 'in vitro fertilizations':ti,ab,kw OR ivf:ti,ab,kw

#3 'sperm injections, intracytoplasmic':ti,ab,kw OR icsi:ti,ab,kw OR 'injection, intracytoplasmic sperm':ti,ab,kw OR 'injections, intracytoplasmic sperm':ti,ab,kw OR 'intracytoplasmic sperm injection':ti,ab,kw OR 'sperm injection, intracytoplasmic':ti,ab,kw OR 'intracytoplasmic sperm injections':ti,ab,kw OR 'injections, sperm, intracytoplasmic':ti,ab,kw OR 'assisted reproductive technology':ti,ab,kw

#4 #1 OR #2 OR #3

#5 'endometrial compaction':ti,ab,kw OR 'endometrial thickness change':ti,ab,kw OR 'change, endometrial thickness':ti,ab,kw OR 'endometrial thickness decreased':ti,ab,kw OR 'endometrial thickness compacted':ti,ab,kw

#6 'pregnancy outcome':ti,ab,kw OR 'pregnancy outcomes':ti,ab,kw OR 'outcome, pregnancy':ti,ab,kw OR 'outcomes, pregnancy':ti,ab,kw OR 'clinical outcomes':ti,ab,kw OR 'live birth':ti,ab,kw OR 'clinical pregnancy':ti,ab,kw OR 'ongoing pregnancy':ti,ab,kw

#7 #4 AND #5 AND #6

WEB OF SCIENCE:168

**#1**

**TS=(Embryo Transfer OR Embryo Transfers OR Transfer, Embryo OR Transfers, Embryo OR Blastocyst Transfer OR Blastocyst Transfers)**

**#2**

**TS=(Fertilizations in Vitro OR In Vitro Fertilization OR In Vitro Fertilizations OR IVF)**

**#3**

**TS=(Injection, Intracytoplasmic Sperm OR Injections, Intracytoplasmic Sperm OR Intracytoplasmic Sperm Injection OR Sperm Injection, Intracytoplasmic OR Intracytoplasmic Sperm Injections OR ICSI OR Injections, Sperm, Intracytoplasmic)**

**#4**

**#1 OR #2 OR #3**

**#5**

**TS=(Endometrial compaction OR Endometrial thickness change OR change, endometrial thickness OR Endometrial thickness decreased OR Endometrial thickness compacted)**

**#6**

**TS=(Pregnancy Outcomes OR Outcome, Pregnancy OR Outcomes, Pregnancy OR Clinical outcomes OR Live birth OR Clinical pregnancy OR Ongoing pregnancy)**

**#7**

**#4 AND #5 AND #6**
