# Supplementary material for: Does endometrial compaction before embryo transfer affect pregnancy outcomes? a systematic review and meta-analysis
Source: Front Endocrinol (Lausanne). 2023 Nov 14;14:1264608. doi: 10.3389/fendo.2023.1264608 (PMC10682779; doi:10.3389/fendo.2023.1264608)
Supplement: Supplementary Appendix 2 — Newcastle-Ottawa Scale [file Table_2.docx]

| **Study** | **Selection** | | | | **Comparability** | **Outcome** | | | **Quality score** |
| --- | --- | --- | --- | --- | --- | --- | --- | --- | --- |
|  | **Representativeness of the exposed cohort** | **Selection of the non-exposed cohort** | **Ascertainment of exposure** | **Demonstration that outcome of interest was not present at start of study** | **Comparability of cohorts on the basis of the design or analysis** | **Assessment of outcome** | **Was follow-up long enough for outcomes to occur** | **Adequacy of follow up of cohorts** |  |
| **Haas 2019** | **b** | **a** | **a** | **a** | **b** | **b** | **b** | **a** | **7** |
| **Bu 2019** | **b** | **a** | **a** | **a** | **a, b** | **a** | **b** | **a** | **8** |
| **Zilberberg 2020** | **c** | **a** | **a** | **a** | **b** | **b** | **b** | **a** | **6** |
| **Huang 2020** | **b** | **a** | **a** | **a** | **a, b** | **b** | **a** | **a** | **9** |
| **Ye 2020** | **b** | **a** | **a** | **a** | **a, b** | **b** | **a** | **a** | **9** |
| **Huang 2021** | **b** | **a** | **a** | **a** | **a, b** | **b** | **a** | **a** | **9** |
| **Riestenberg 2021** | **c** | **a** | **a** | **a** | **a, b** | **a** | **a** | **a** | **8** |
| **Al Jarrah 2021** | **c** | **a** | **a** | **a** | **a, b** | **a** | **b** | **a** | **7** |
| **Yaprak 2021** | **b** | **a** | **a** | **a** | **b** | **b** | **a** | **a** | **8** |
| **Jin（a）2021** | **c** | **a** | **a** | **a** | **a, b** | **b** | **a** | **a** | **8** |
| **Lam 2021** | **b** | **a** | **a** | **a** | **a, b** | **b** | **a** | **a** | **9** |
| **Jin（b）2021** | **c** | **a** | **a** | **a** | **a, b** | **b** | **a** | **a** | **8** |
| **Kaye 2021** | **b** | **a** | **a** | **a** | **a, b** | **b** | **b** | **a** | **8** |
| **Shah 2022** | **c** | **a** | **a** | **a** | **b** | **a** | **a** | **a** | **7** |
| **Youngster 2022** | **c** | **a** | **a** | **a** | **a, b** | **a** | **a** | **a** | **8** |
| **Olgan 2022** | **b** | **a** | **a** | **a** | **a, b** | **a** | **b** | **a** | **8** |
| **Gursu2022** | **c** | **a** | **a** | **a** | **a, b** | **b** | **a** | **a** | **8** |
| **Jafarabadi2023** | **c** | **a** | **a** | **a** | **a, b** | **b** | **a** | **a** | **8** |
| Jin（a）：Jin, Z., et al., *Endometrial thickness changes after progesterone administration do not affect the pregnancy outcomes of frozen-thawed euploid blastocyst transfer: a retrospective cohort study.* Fertil Steril, 2021. 116(6): p. 1502-1512.  Jin（b）：Jin, Z.Q., et al., *Effect of endometrial thickness changes on clinical pregnancy rates after progesterone administration in a single frozen-thawed euploid blastocyst transfer cycle using natural cycles with luteal support for PGT-SR- and PGT-M-assisted reproduction: a retrospective cohort study.* REPRODUCTIVE BIOLOGY AND ENDOCRINOLOGY, 2021. **19**(1). | | | | | | | | | |

**COHORT STUDIES**

Note: A study can be awarded a maximum of one star for each numbered item within the Selection and Outcome categories. A maximum of two stars can be given for Comparability

**Selection**

1) Representativeness of the exposed cohort

a) truly representative of the average _______________ (describe) in the community **¯**

b) somewhat representative of the average ______________ in the community **¯**

c) selected group of users eg nurses, volunteers

d) no description of the derivation of the cohort

2) Selection of the non exposed cohort

a) drawn from the same community as the exposed cohort **¯**

b) drawn from a different source

c) no description of the derivation of the non exposed cohort

3) Ascertainment of exposure

a) secure record (eg surgical records) **¯**

b) structured interview **¯**

c) written self report

d) no description

4) Demonstration that outcome of interest was not present at start of study

a) yes **¯**

b) no

**Comparability**

1) Comparability of cohorts on the basis of the design or analysis

a) study controls for _____________ (select the most important factor) **¯**

b) study controls for any additional factor **¯** (This criteria could be modified to indicate specific control for a second important factor.)

**Outcome**

1) Assessment of outcome

a) independent blind assessment **¯**

b) record linkage **¯**

c) self report

d) no description

2) Was follow-up long enough for outcomes to occur

a) yes (select an adequate follow up period for outcome of interest) **¯**

b) no

3) Adequacy of follow up of cohorts

a) complete follow up - all subjects accounted for **¯**

b) subjects lost to follow up unlikely to introduce bias - small number lost - > ____ % (select an adequate %) follow up, or description provided of those lost) **¯**

c) follow up rate < ____% (select an adequate %) and no description of those lost

d) no statement
