# Supplementary figures and images for: Does endometrial compaction before embryo transfer affect pregnancy outcomes? a systematic review and meta-analysis
Source: Front Endocrinol (Lausanne). 2023 Nov 14;14:1264608. doi: 10.3389/fendo.2023.1264608 (PMC10682779; doi:10.3389/fendo.2023.1264608)

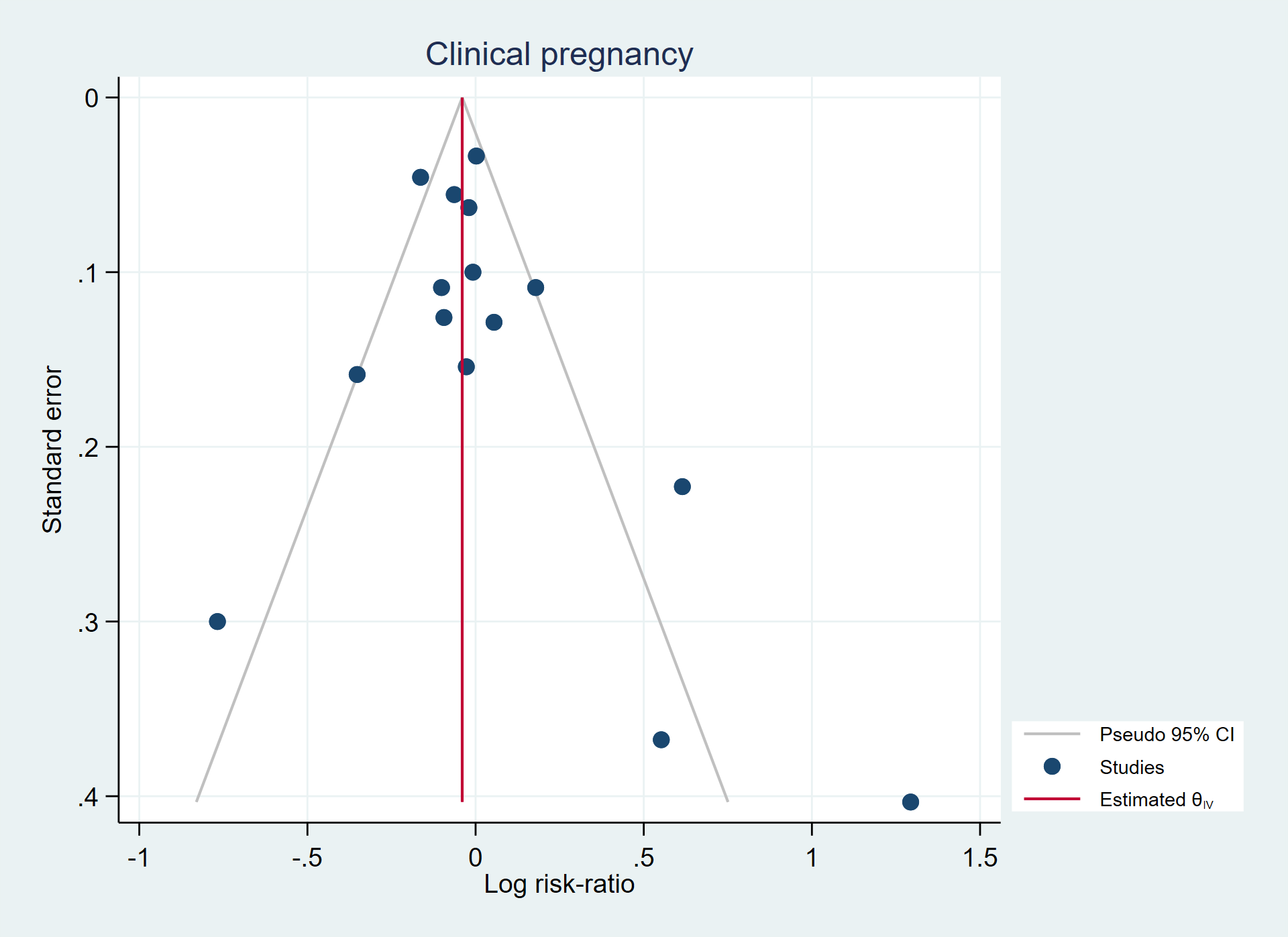

Supplement: Supplementary Appendix 3-1 — Funnel plot of clinical pregnancy. [file Image_1.png]

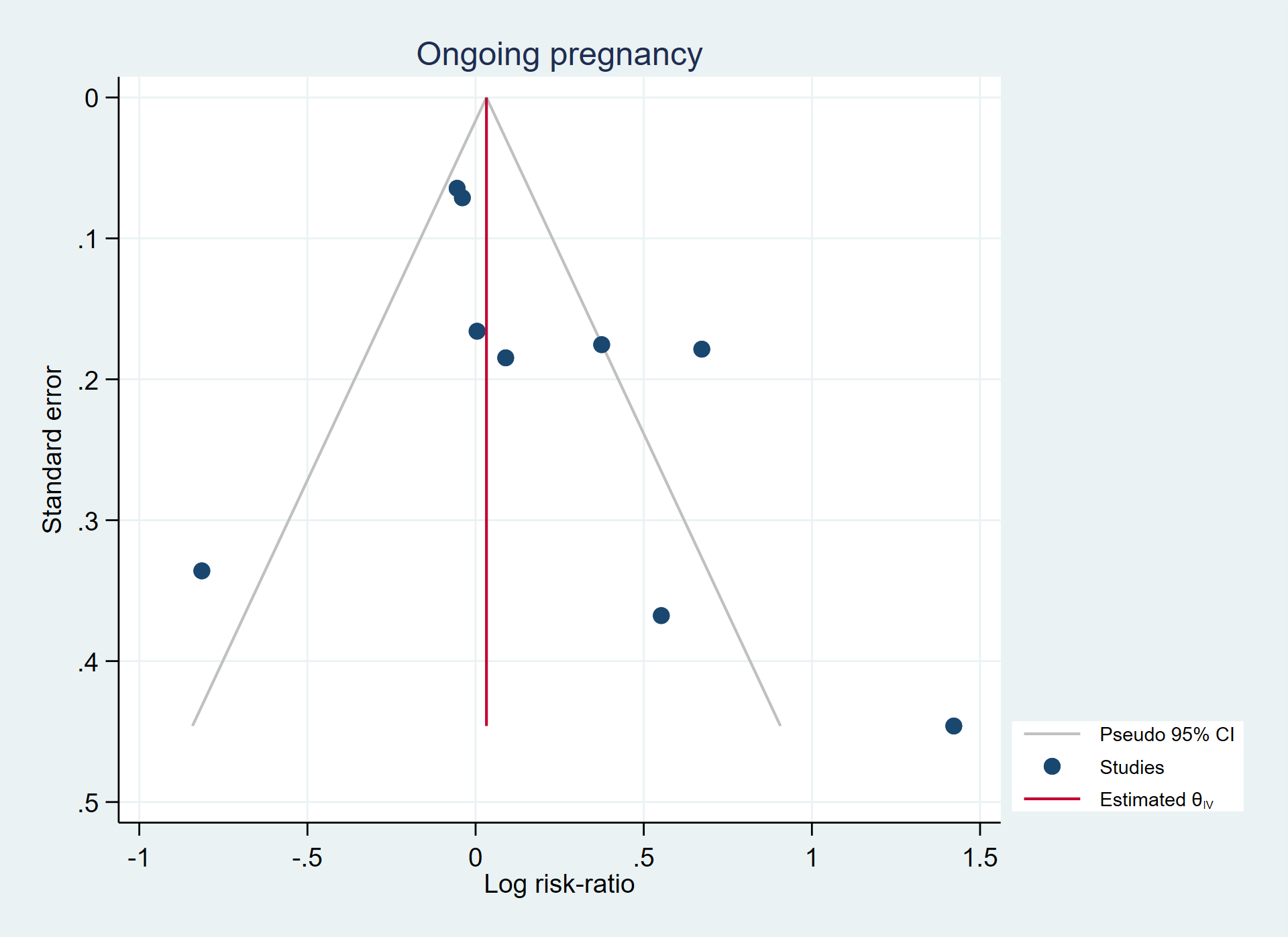

Supplement: Supplementary Appendix 3-2 — Funnel plot of ongoing pregnancy. [file Image_2.png]

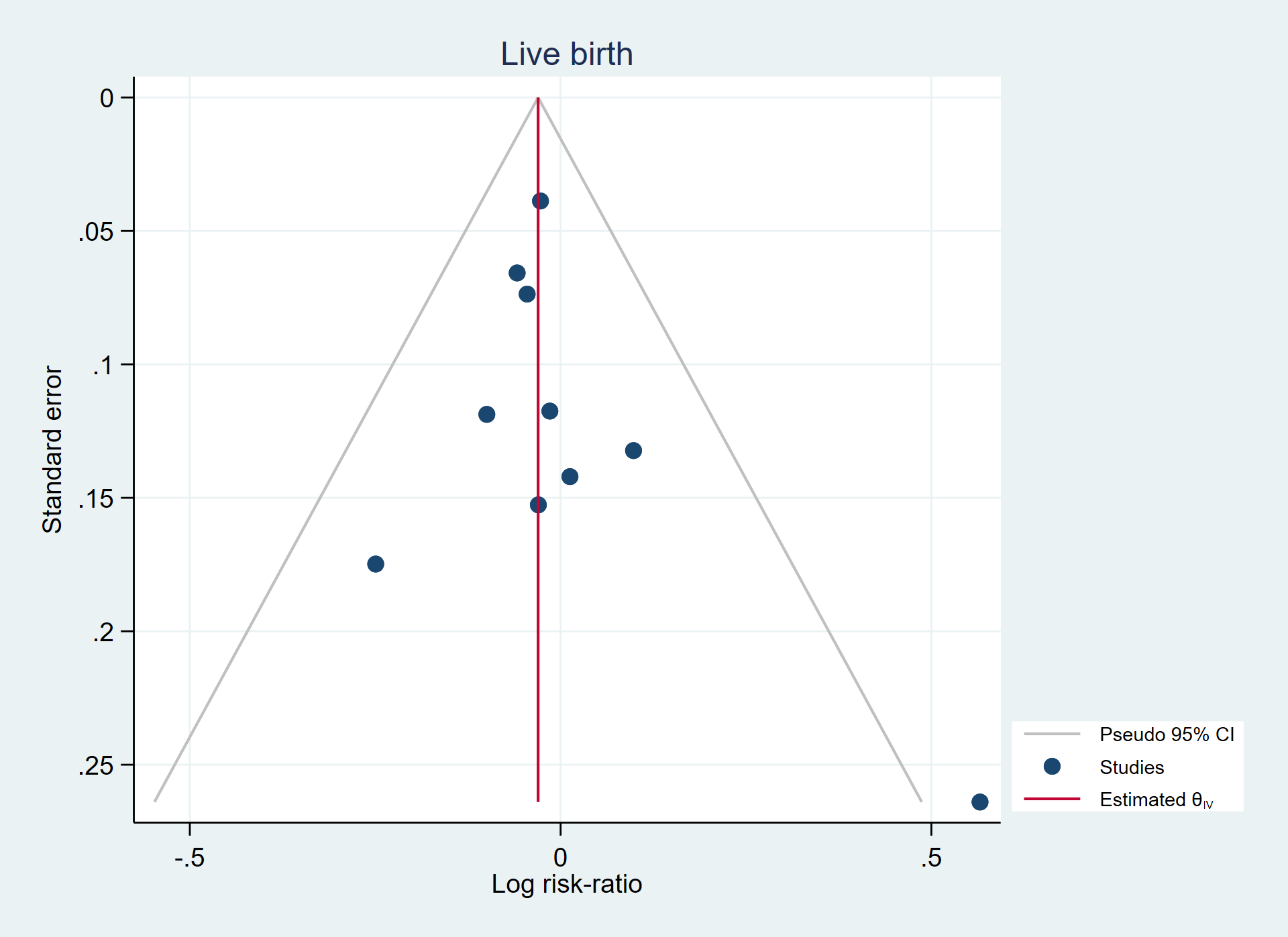

Supplement: Supplementary Appendix 3-3 — Funnel plot of live birth. [file Image_3.png]

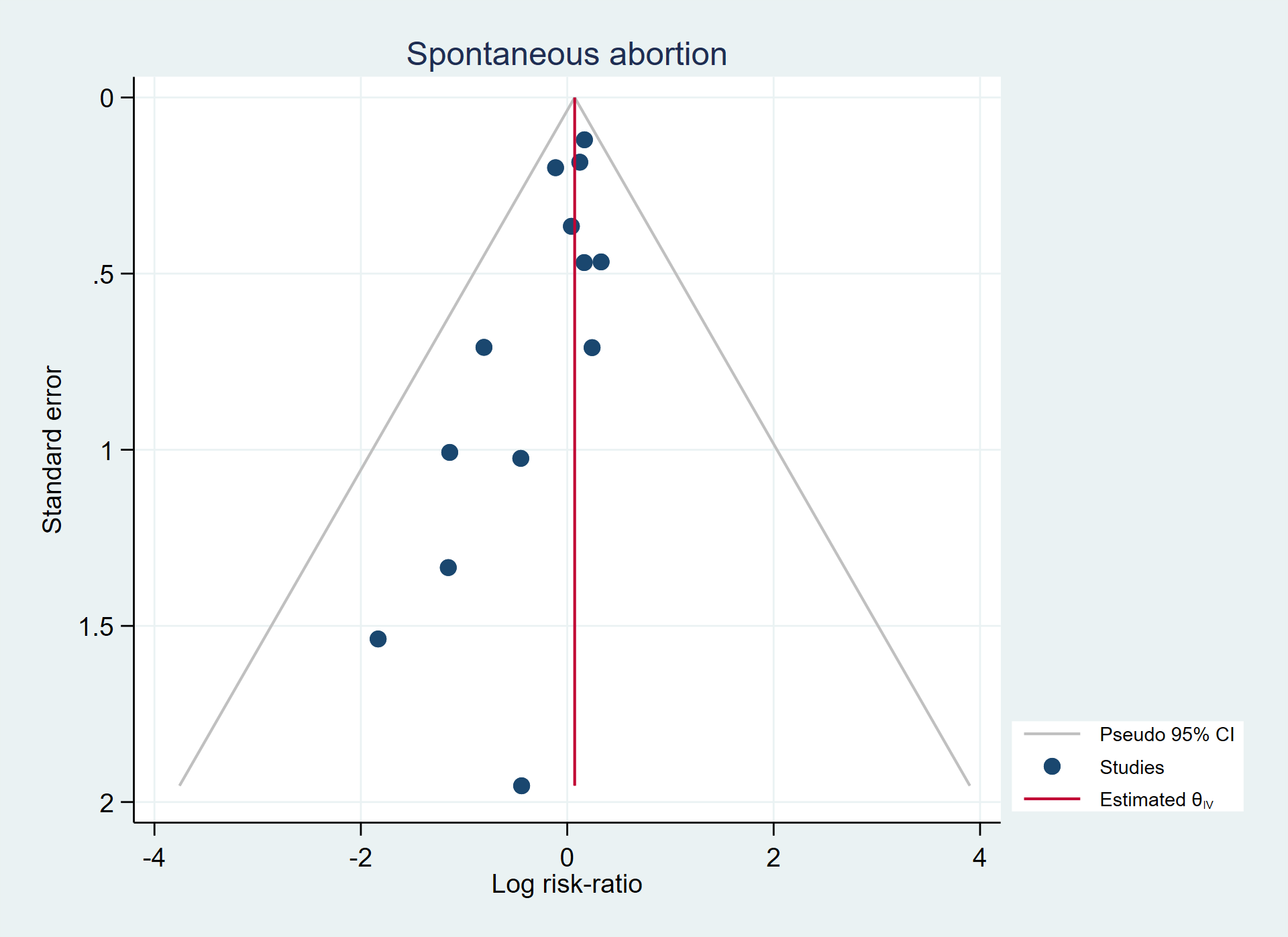

Supplement: Supplementary Appendix 3-4 — Funnel plot of spontaneous abortion. [file Image_4.png]
